# Sustained interleukin-10 delivery reduces inflammation and improves motor function after spinal cord injury

**DOI:** 10.1186/s12974-019-1479-3

**Published:** 2019-04-30

**Authors:** Daniel J. Hellenbrand, Kaitlyn A. Reichl, Benjamin J. Travis, Mallory E. Filipp, Andrew S. Khalil, Domenic J. Pulito, Ashley V. Gavigan, Elizabeth R. Maginot, Mitchell T. Arnold, Alexander G. Adler, William L. Murphy, Amgad S. Hanna

**Affiliations:** 1 Department of Neurological Surgery, University of Wisconsin, Madison, WI 53792 USA; 2 Department of Biomedical Engineering, University of Wisconsin, Madison, WI 53706 USA; 3 Department of Orthopedics and Rehabilitation, University of Wisconsin, Madison, WI 53705 USA

**Keywords:** Interleukin-10, Macrophages, Microglia, Spinal cord injury, Inflammation

## Abstract

**Background:**

The anti-inflammatory cytokine interleukin-10 (IL-10) has been explored previously as a treatment method for spinal cord injury (SCI) due to its ability to attenuate pro-inflammatory cytokines and reduce apoptosis. Primary limitations when using systemic injections of IL-10 are that it is rapidly cleared from the injury site and that it does not cross the blood–spinal cord barrier.

**Objective:**

Here, mineral-coated microparticles (MCMs) were used to obtain a local sustained delivery of IL-10 directly into the injury site after SCI.

**Methods:**

Female Sprague-Dawley rats were contused at T10 and treated with either an intraperitoneal injection of IL-10, an intramedullary injection of IL-10, or MCMs bound with IL-10 (MCMs+IL-10). After treatment, cytokine levels were measured in the spinal cord, functional testing and electrophysiology were performed, axon tracers were injected into the brainstem and motor cortex, macrophage levels were counted using flow cytometry and immunohistochemistry, and lesion size was measured.

**Results:**

When treated with MCMs+IL-10, IL-10 was significantly elevated in the injury site and inflammatory cytokines were significantly suppressed, prompting significantly less cells expressing antigens characteristic of inflammatory macrophages and significantly more cells expressing antigens characteristic of earlier stage anti-inflammatory macrophages. Significantly more axons were preserved within the rubrospinal and reticulospinal tracts through the injury site when treated with MCMs+IL-10; however, there was no significant difference in corticospinal tract axons preserved, regardless of treatment group. The rats treated with MCMs+IL-10 were the only group with a significantly higher functional score compared to injured controls 28 days post-contusion.

**Conclusion:**

These data demonstrate that MCMs can effectively deliver biologically active IL-10 for an extended period of time altering macrophage phenotype and aiding in functional recovery after SCI.

## Introduction

Activated macrophages are typically described as belonging to two general categories: classically activated M1 macrophages expressing a pro-inflammatory phenotype or alternatively activated M2 macrophages expressing an anti-inflammatory phenotype. However, it is important to note that the M1/M2 paradigm is used as an idealized guide for a dynamic and mixed spectrum of microglia/macrophage activation observed in vivo and antigens from both phenotypes may be expressed, as dictated by the environment. After skin and muscle injuries, there is sequential activation of classically activated pro-inflammatory M1 macrophages and alternatively activated M2a, M2b, and M2c macrophages, which facilitates in transitioning through inflammatory, proliferative, and remodeling phases of repair. After spinal cord injury (SCI), this sequential activation does not occur and pro-inflammatory macrophages potentiate a prolonged inflammation phase similar to what is observed in chronic non-healing wounds [1–4]. Although prolonged inflammation is detrimental, the immune cells instigated by the production of pro-inflammatory cytokines aid in preventing infection by phagocytosing bacteria, viruses, and foreign bodies near the injury site [5]. This emphasizes the importance of the inflammatory process and that SCI treatment may involve adjusting the timeline of M1 macrophages rather than inhibiting them.

M2 macrophages function in wound healing by producing anti-inflammatory cytokines, such as Interleukin-10 (IL-10), and aiding in the conversion of M1 macrophages to the M2 phenotype [6, 7]. Many studies performed over the past few decades have revealed the positive effects of IL-10 treatment post-SCI [8]. IL-10 treatments have been shown to protect against secondary inflammation by downregulating pro-inflammatory cytokines [6, 9, 10], provide trophic support and neuroprotection [11, 12], and improve functional recovery in rat models [13–15]. However, research in IL-10 treatments has identified key obstacles, such as the rapid clearing of IL-10 from the injury site [16] and its inability to cross the blood–spinal cord barrier [17].

We hypothesized a sustained release of IL-10 directly in the injury site would induce the conversion of pro-inflammatory macrophages to an anti-inflammatory phenotype that decreases apoptosis and encourages tissue repair, thus preserving axons through the injury site and improving hind limb function after SCI. Here, we used mineral-coated microparticles (MCMs) to obtain a sustained delivery of IL-10 directly in the injury site.

## Materials and methods

### Experimental design and statistical analysis

**Fig. 1:**
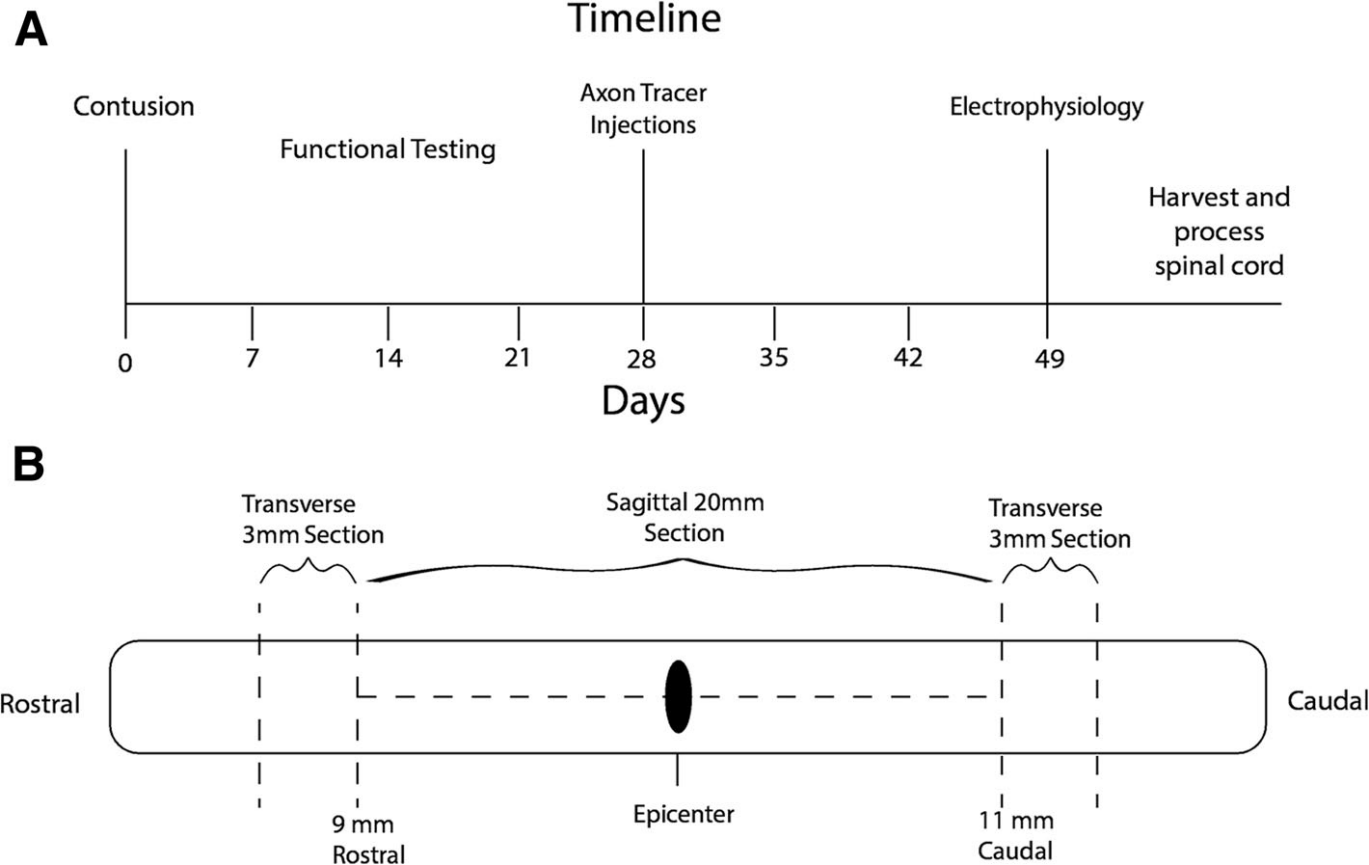
The experimental timeline indicating when each procedure was performed, from the initial contusion to spinal cord harvesting (**a**). A diagram displaying the segments of spinal cord used for analysis (**b**). The two 3 mm segments were sectioned transversely and used to count labeled axons both rostral and caudal to the injury. The 20-mm segment containing the epicenter was sectioned sagittally and used to quantify macrophage levels and measure the lesion size

The surgical procedures were performed following the NIH Guide for the care and use of laboratory animals and in accordance with protocols approved by the University of Wisconsin—Madison Animal Use & Care Committee. The experimental design followed the timeline presented in Fig. 1a, using female Sprague-Dawley rats with an approximate weight and age of 225–250 g and 2 months, respectively (Sprague Dawley; Envigo, Huntingdon, UK). All rats were housed 2 per cage at room temperature, standard light/dark cycle with food and water at lib.

The experiment utilized five test groups, to which rats were randomly assigned (Table 1). Prior to inclusion in the study, the rats were assessed using the Basso-Beattie-Bresnahan (BBB) locomotor scale to ensure there were no motor deficits. All groups underwent a spinal cord contusion injury at T10. The treatments compared were as follows: Control group, Systemic IL-10 group, Local IL-10 group, MCMs group, and MCMs+IL-10 group. Following the impaction, each group, excluding the Controls, received therapeutic intervention as described in Table 1. To determine group sizes, a power of analysis was conducted with 80% power and a significance level (alpha) of 0.05 (Student’s *t* test two-tailed, StatMate). For measuring the level of cytokines, a sample size of seven in each group at each test point (24 h and 7 days) has 80% power to detect a difference between means of 20% and for functional testing a sample size of 10 in each group has 80% power to detect a difference between mean BBB scores of two.

**Table 1:** Descriptions of treatments tested including timing, dosage, and location

Groups	Treatments
Control	T10 contusion, no treatment
Systemic IL-10	50 μl IL-10 (concentration 5 μg IL-10/50 μl 1X PBS) via IP injection 30 min post-impaction
Local IL-10	5 μl IL-10 (concentration 10 μg/1 ml 1X PBS) injected into the epicenter, 1 μL at a time in 30-s intervals
MCMs	5 μl MCMs (concentration 20 mg MCMs/1 ml 1X PBS) injected into the epicenter, 1 μL at a time in 30-s intervals
MCMs+IL-10	5 μl MCMs bound with IL-10 (concentration 20 mg of IL-10 MCMs/1 ml 1X PBS) injected into the epicenter, 1 μL at a time in 30-s intervals

Contused rats refers to all groups: Controls, Systemic IL-10, Local IL-10, MCMs, and MCMs+IL-10

All statistical analyses were performed in Prism 6 (GraphPad Software, San Diego, CA). Functional scores were analyzed using two-way ANOVA with days as the repeated measures; one-way ANOVA was used to analyze cytokine levels, axon counting, electrophysiology, macrophage levels, and lesion size. If results were deemed significant, Tukey’s post-hoc analysis was used to test between groups or Dunnett’s post-hoc analysis was used to compare treatments to Controls. Differences were considered significant at *P* < 0.05. Quantitative data are presented as mean ± standard error of the mean (SEM).

### Development of mineral-coated microparticles and IL-10 binding

β-tricalcium phosphate (β-TCP) microparticles were obtained from Berkeley Advanced Biomaterials Inc. (Berkeley, CA). In order to form a calcium phosphate mineral coating, the β-TCP microparticles were incubated for 7 days in modified simulated body fluid (mSBF) at 37 °C as previously reported [18–21]. The mSBF has similar ionic strength as human blood plasma with double the concentration of calcium and phosphate. The mSBF was prepared by adding the following reagents into deionized water in the order shown: 141 mM NaCl, 4.0 mM KCl, 0.5 mM MgSO4, 1.0 mM MgCl_2_, 4.2 mM NaHCO_3_, 20.0 mM HEPES, 5.0 mM CaCl_2_, and 2.0 mM KH_2_PO_4_, and the pH was adjusted to 6.80 (Sigma, St. Louis, MO). After incubation in mSBF, the microparticles were strained through a 40-μm screen and the morphology and composition of the calcium phosphate mineral coating on the microparticles were examined, after sputter coating with gold, by scanning electron microscopy and energy dispersive spectroscopy (EDS; LEO 1530 field emission scanning microscope; Zeiss, Oberkochen, Germany).

For IL-10 binding, 10-mg MCMs were incubated in 500 μL of 1X PBS containing 2 μg Recombinant Rat IL-10 (Peprotech, Rocky Hill, NJ, Cat# 400–19) for 4 hours at 37 °C. An in vitro test to determine efficacy of the sustained release of IL-10 from the MCMs was performed by incubating the MCMs with IL-10 bound in simulated body fluid at physiological conditions. The MCMs were switched to a new tube every other day and the amount of IL-10 released into solution was measured using an enzyme-linked immuno-sorbent assay kit (ELISA; Abcam Cambridge, UK; Cat# ab100765).

### Spinal cord injury

A laminectomy was completed at T10 exposing the spinal cord, and the rat was stabilized and slightly elevated off the platform by clamps on the T9 and T11 processes, after which a MASCIS Weight Drop machine (WM Keck Center for Collaborative Neuroscience, Rutgers University, Piscataway, NJ; Model II) was used to perform a 10-g weight drop from a height of 12.5 mm. Immediately after impaction, the injections into the epicenter of the injury were completed at a depth of 1.5 mm at a rate of 1 μl every 30 s. The anesthetic agent used during this procedure was 16:1 ketamine/xylazine mixture via intraperitoneal injection.

Immediately following the completion of the SCI procedure, the rats received subcutaneous injections of 0.05 mg/kg buprenorphine and 10 mg/kg enrofloxacin to control pain and prevent infections, respectively. They also received an antibiotic-enriched diet, Uniprim (Envigo, Huntingdon, United Kingdom; Cat# TD.06596), for 1 week following SCI. The rats’ bladders were expressed twice daily until function was regained. Animals with urinary tract infections were treated for 5 days with 10 mg/kg enrofloxacin once a day, subcutaneously. The efficacy of the SCI was determined on day one post-operation through BBB functional testing; a day one baseline BBB score of three or higher indicated an atypical injury, prompting immediate exclusion from the study.

### Cytokine levels in the spinal cord

Cytokine levels were measured at 24 h and 7 days after SCI. After the predetermined waiting period, the rats were given a lethal dose of isoflurane and their blood was flushed out with 0.9% saline transcardially. Immediately after flushing the blood, a 4-mm section of the spinal cord centered at the epicenter of the injury site was taken and weighed. The section of spinal cord was then minced using a scalpel and homogenized. The homogenates were placed in lysate buffer and centrifuged at 12,000×*g* for 15 min at 4 °C. The supernatants were removed, aliquoted on ice, and placed in a − 80 °C freezer. To quantify cytokine levels, ELISA kits for detecting IL-10, tumor necrosis factor-alpha (TNFα), and interluekin-1 beta (IL-1β) were used and performed according to manufacturer’s directions (ELISA kit; Abcam Cambridge, UK; IL-10: Cat# ab100765, TNFα: Cat# ab100785, IL-1β: Cat# ab100768). The results for each group are presented as the percent increase from uninjured rats.

### Macrophage phenotype

#### Flow cytometry

Seven days post-SCI, the rats were given a lethal dose of isoflurane and their blood was flushed out with 0.9% saline transcardially. Immediately after flushing the blood, a 4-mm section of the spinal cord centered at the epicenter of the injury site was harvested. The excised tissue was mechanically digested for 5 min using a scalpel prior to incubation in 1 mL of a fivefold dilution of 10 × collagenase/hyaluronidase in DMEM (StemCell Technologies Cat# 07912) for 30 min at 37 °C on a rotary mixer to which 200 Units/mL of DNase I (Sigma-Aldrich Cat# DN25-1G) was added. The enzymatic digest was titrated 20 × with a 1-mL micropipette and followed by addition of 1 mL of DMEM containing 10% FBS. The cells were centrifuged at 500×*g* for 5 min and washed 2 times with PBS and strained through a 100-μm cell strainer on the last wash. The cells were incubated in 1 μL/mL of Ghost Dye Red 780 live/dead (TonboBio Cat# 13–0865-T100) for 30 min at 4 °C protected from light. PBS containing 10% FBS was added to the live/dead stain after 30 min and the cells were centrifuged at 500×*g* for 5 min following a second wash with PBS containing 10% FBS. The inclusion of 10% FBS was used to block non-specific binding sites [22–24]. The cells were resuspended in 1% formaldehyde in PBS and incubated for 20 min at room temperature protected from light. The fixed cells were centrifuged at 500×*g* for 5 min and washed once with PBS. The cells were then resuspended in ice cold 90% methanol in PBS for storage until labeling for flow cytometry.

Cells were labeled for all macrophages using CD11b and CD45. Ramified microglia are CD11b^+^ CD45^LOW^ and fully activated macrophages are CD11b^+^ CD45^HIGH^ [25, 26]. M1-specific markers CD68 and CD80, M1 or M2b marker CD86, and M2c marker CD163 were used to further distinguish macrophage phenotype on the CD11b^+^ CD45^HIGH^ cell population [1, 27]. Eight samples selected at random were pooled and counted using a hemocytometer to determine the average cell isolation yield. This yield average was used to estimate the cell isolation concentrations and approximately 250,000 cells were used for antibody labeling and subsequent flow cytometry analysis. The cells were added to a 96-well polypropylene v-bottom plate and washed 2 times with 0.5% BSA in PBS, referred to as flow bluffer 1 (FB1), to remove the 90% methanol storage solution. The cells were then incubated in 100 μL of primary antibody solutions at 1:10 dilutions for CD11b-Pacific Blue (Bio-Rad/AbD Serotec Cat# MCA275PB, RRID:AB_566459), CD163-Biotin (Bio-Rad/AbD Serotec Cat# MCA342B, RRID:AB_2074559), CD68-PE (Bio-Rad/AbD Serotec Cat# MCA341PE, RRID:AB_324585), CD86-Alexafluor 647 (Bio-Rad/AbD Serotec Cat# MCA2874A647, RRID:AB_1719961), and CD45-PE/Alexafluor 750 (Bio-Rad/AbD Serotec Cat# MCA43P750, RRID:AB_10673436) and 1:1 dilution for CD80-FITC (LS Bio Cat#C188420–100) in PBS containing 0.5% FBS and 0.1% TritonX-100, referred to as flow buffer 2 (FB2), for 1 h at room temperature and protected from light. The primary-labeled cells were washed 2 times with FB2 and then incubated in 100 μL of secondary antibody at 1:2000 dilution for Streptavidin-Alexafluor 430 (Thermo Fisher Scientific Catalog #S11237) in FB2 (or just 100 μL FB2 with no secondary for the primary-fluorophore conjugates) for 30 min at room temperature protected from light. The fully labeled cells were washed 2 times in FB2 and resuspended in FB1 for flow cytometry analysis. Aliquots from 8 samples were additionally pooled and labeled with the above protocol containing all primaries minus one for each antigen, referred to as fluorescence minus one (FMO). Each primary (and corresponding secondary if applicable) were also individually added to 1 drop of UltraComp eBeads (Thermo Fisher Scientific Catalog #01–2222-41) at the cell labeling concentrations for fluorescence signal compensation. Each sample, compensation control, and FMO were run on an Attune Nxt (Thermo Fisher Scientific) and 10,000 single and live cell events were collected. After application of compensation to the sample data, a positive gate for each antigen was determined as sample fluorescence above ~ 1% positive expression in the appropriate FMO for single live-stained cells.

#### Macrophage labeling and quantification

Seven weeks post injury, spinal cords were harvested and paraffin embedded. Sagittal segments were sectioned 10-μm thick and placed on slides. Sections were deparaffinized and rehydrated. A 1:100 antigen unmasking solution in distilled water was used to expose antigens (Vector Laboratories Cat# H-3300, RRID: AB_2336226). The slides were then washed with 0.1 M PBS for 5 min, followed by 1 h in nDS blocker (4% nDS, 1% BSA, 0.5% Triton, 0.1 M PBS). The primary staining solution consisted of 10 μg/mL biotinylated tomato lectin (TL) (Vector Laboratories Cat# B-1175, RRID: AB_2315475), 1:100 mouse-anti-CD163 (Bio-Rad/AbD Serotec Cat# MCA342GA, RRID: AB_2074558), and 1:100 rabbit-anti-MARCO (macrophage receptor with collagenous structure) (Abcam Cat# ab108113, RRID: AB_10861943) combined with 25% blocker and 75% 0.1 M PBS. The slides were submerged in the primary solution for 24 h at 4 °C. The slides were then rinsed with 0.1 M PBS. A secondary solution composed of Streptavidin 350 (Thermo Fisher Scientific Cat# S11249), donkey-anti-mouse 594 (Thermo Fisher Scientific Cat# A-21203, RRID: AB_2535789), and donkey-anti-rabbit 488 (Thermo Fisher Scientific Cat# A-21206, RRID: AB_2535792) all in a 1:500 ratio with 0.1 M PBS was applied to the slides for 1 h at room temperature. The slides were again rinsed with 0.1 M PBS and cover slipped with Prolong Gold without DAPI (Thermo Fisher Scientific Cat# P36930).

Five rats from each group were analyzed. All sections were imaged with a Keyence BZ-9000 fluorescence microscope (Keyence, Osaka, Japan). To quantify macrophages, a × 20 image of the injury site was taken in three sections from each rat and the percent of area stained was calculated in each image using ImageJ (NIH, Bethesda, MD).

### Functional testing

Functional recovery was assessed weekly for 4 weeks post-injury through visual observation using the BBB locomotor rating scale. During the assessment, the rats were continuously videotaped for a period of 4 min in an open field. The BBB scale, a functional analysis, was used to evaluate rats with SCI rating them from 0 to 21 based on a combination of limb and joint movement, limb coordination, stability, tail position, and abdomen position [28]. Individuals blinded to the treatment groups completed all scoring.

### Axon tracer injections and axon counting

Four weeks after SCI, seven rats from each group underwent anterograde axon tracing with a 10% biotinylated dextran amine (BDA) (Thermo Fisher Scientific Cat# D1956, RRID: AB_2307337) in saline, as previously reported [29]. Three of the seven rats received BDA injections in eight sites of the motor cortex (MC). The following coordinates were used to inject 1 μl BDA per site at a rate of 0.2 μl every 30 s, using the bregma as the zero point: anterior–posterior (AP), 1 mm; medial–lateral (ML), ± 1.8 mm; dorsal–ventral (DV, from dural surface), 1.0 mm; AP, 0 mm; ML, ± 2 mm; DV, 1 mm; AP, + 1 mm; ML, ± 2.2 mm; DV, 1 mm; AP, +  2 mm; ML, ± 2.6 mm; DV, 1 mm. The remaining four rats received BDA injections into the red nucleus (RN) and reticular formation (RF) of the brainstem, 0.5 μl BDA per site at a rate of 0.1 μl every 30 s. Using the bregma as the zero point, the following coordinates were used: RN: AP, 5.8 mm; ML, ± 1.2 mm; DV, 7 mm; RF: AP, 11.6 mm; ML, ± 1 mm; DV, 6 mm. For both the brainstem and MC injections, the needle remained in place for 1 min post-injection to allow for tissue absorption. The anesthetic agent used for this procedure was 4% isoflurane in oxygen to anesthetize the rats and maintained for the duration of the surgery at 2–3% isoflurane in oxygen. The MC and brainstem-injected rats were maintained alive for an additional 2 and 3 weeks, respectively, post-injection. They then received a lethal dose of isoflurane, were perfused with 0.9% saline followed by 4% paraformaldehyde (PFA; Sigma; Cat# 441244) in 0.1 M PBS, pH 7.4, transcardially, and the spinal cords were harvested.

The harvested cords were submerged in 4% PFA for 24 h and then parsed into three sections: two 3-mm sections taken 11-mm caudal and 9-mm rostral to the epicenter of the injury site, and the remaining 20-mm section containing the injury site (Fig. 1b). The two 3-mm sections were placed into a 30% sucrose solution (Sucrose, Sigma, Cat# S7903) for 48 h, frozen in Tissue-Tek® and sectioned transversely at a thickness of 20 μm. The 20 mm section containing the epicenter was cut sagittally along the ventral split, dehydrated in alcohol, embedded in paraffin, and sectioned 10-μm thick for macrophage counting and lesion size analysis.

Slides containing transverse sections of spinal cord from rostral and caudal to the injury site, as shown in Fig. 1b, were rinsed with 0.1 M PBS for 10 min, followed by 1 hour in normal donkey serum (nDS) blocker (4% nDS, 1% bovine serum albumin (BSA), 0.5% Triton, 0.1 M PBS). The slides were then submerged in the secondary solution consisting of a 1:500 ratio of Streptavidin 594 (Thermo Fisher Scientific Cat# S-11227, RRID: AB_2313574) to 0.1 M PBS for 2 hours in the dark at room temperature. Then, the slides were rinsed with 0.1 M PBS and cover slipped using Prolong Gold with 4′,6-diamidino-2-phenylindole (DAPI) (Thermo Fisher Scientific Cat# P36930).

Three 20-μm transverse sections from each the rostral 3-mm segment and the caudal 3-mm segment were used. The average of the three rostral sections was considered the total number of axons labeled in each rat, with the average of the three caudal sections representing the number of labeled axons crossing the injury site. All sections were imaged on a Keyence BZ-9000 fluorescence microscope (Keyence, Osaka, Japan). Using the Keyence Analyzer software, a region of interest was drawn excluding the gray matter and the numbers of axons with a size between 1 and 15 μm in diameter were quantified.

### Electrophysiology

Rats were anesthetized with 16:1 ketamine/xylazine mixture via intraperitoneal injection. Three rats from each group underwent electrophysiology testing to measure the cord dorsum potential (CDP). The spinal cord was exposed by means of a laminectomy at the T8 and T11 levels. Clamps were placed on the spinous processes of T7 and T12 to stabilize and elevate the rat. Upon opening the dura at T8, the cathode stimulating monopolar microelectrode (Microprobes for Life Sciences, Gaithersburg, MD, Cat# PI20030.5A10) was placed at a depth of 0.9 mm below the dural surface, and the recording hook electrode (NeuroSign, The Magstim Company Limited, Wales, UK, Cat# 4009–00) rested gently on top of the intact dura at the T11 level, approximately 15-mm caudal to the stimulating electrode (Fig. 11a). The anode needle for stimulation was placed in the muscle directly adjacent to the microelectrode.

A constant current stimulus isolator (World Precicion Instruments Model A385) was used to send a 400-μA current for a duration of 25 μs, the signal was amplified (Grass Model 7P511) and the average of 30 pulses was read into LabView. The conduction velocity was calculated from the distance between the stimulating and recording electrode divided by the time between the stimulus and the CDP. The amplitude was measured from peak-to-peak.

### Lesion size analysis

The lesion size was determined using the sagittal 20-mm paraffin embedded spinal cord segments (Fig. 1b), which were sectioned 10-μm thick. Sections were deparaffinized and rehydrated. A 1:100 antigen unmasking solution in distilled water was used to expose antigens (Vector Laboratories Cat# H-3300, RRID: AB_2336226). The slides were then washed with 0.1 M PBS, followed by 1 h in nDS blocker (4% nDS, 1% BSA, 0.5% Triton, 0.1 M PBS). The slides were submerged in the primary solution, consisting of a 1:1000 ratio of rabbit-anti-glial fibrillary acidic protein (GFAP; Abcam Cat# ab7260, RRID: AB_305808) combined with 25% blocker and 75% 0.1 M PBS, for 24 h at 4 °C. The slides were then rinsed with 0.1 M PBS. The secondary solution consisting of 1:500 of donkey-anti-rabbit 488 (Thermo Fisher Scientific Cat# A-21206, RRID: AB_2535792) in 0.1 M PBS was applied to the slides for 1 h at room temperature. The slides were again rinsed with 0.1 M PBS and cover slipped with Prolong Gold containing DAPI (Thermo Fisher Scientific Cat# P36930).

Four sections were analyzed per rat, with four rats from each group. In order to properly assess lesion size, two metrics were considered: the amount of spinal atrophy and the size of the infarct. The sectioned tissue was imaged on a Keyence BZ-9000 fluorescence microscope, and quantified using ImageJ software (NIH, Bethesda, Maryland). To determine the level of spinal atrophy present, the diameter of the cord at the epicenter as well as 5-mm rostral and 5-mm caudal to the epicenter were measured. The rostral and caudal measurements were averaged and the epicenter measure was subtracted from the average. To determine the size of infarct, the area of necrotic tissue was measured.

## Results

### MCMs and IL-10 delivery in vitro

**Fig. 2:**
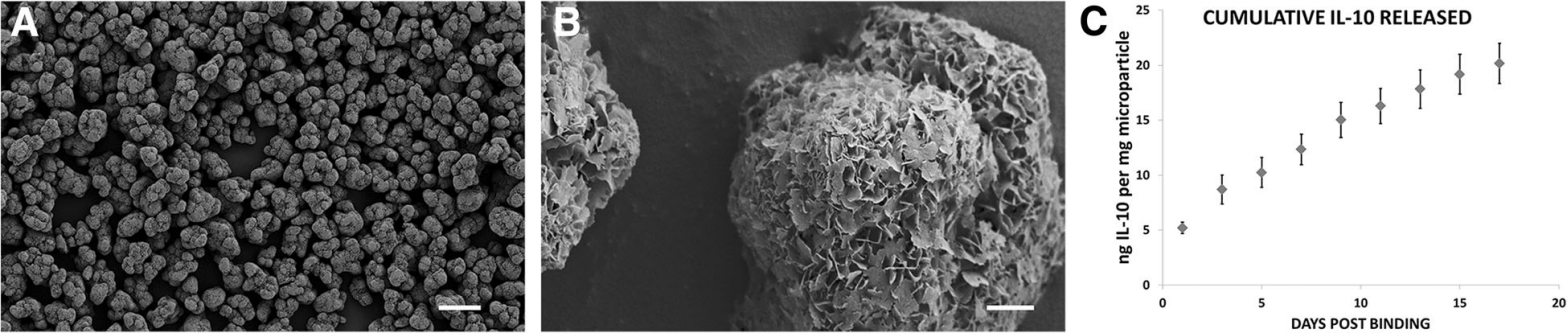
Mineral coated β-TCP microparticles and IL-10 delivery in vitro. After incubation in mSBF, scanning electron microscopy images reveal a continuous plate-like mineral coating covering the entirety of the MCMs (**a, b**), allowing for sufficient binding of IL-10. At physiological conditions in SBF, the release profile of IL-10 from the MCMs was quantified using an ELISA kit. There was an initial burst release of IL-10, followed by a continuous release for at least 17 days (**c**). Scale bars equal 40 μm in **a**, 1 μm in **b**; error bars represent ± SEM in **c**

Following a 7-day incubation in mSBF, scanning electron microscopy revealed an uninterrupted nanoporous plate-like mineral coating covering the entire surface of the MCMs (Fig. 2a, b). Over a course of 17 days, an in vitro release profile of IL-10 from the MCMs was quantified using an ELISA kit. The profile expressed an initial burst release of 5.18 ± 0.5 ng of IL-10 per milligram of MCMs on day one, followed by a linear continuous release for at least 17 days. On day 17 the MCMs with IL-10 bound released 981 ± 270 pg of IL-10 per milligram of MCMs, with a cumulative release of 20.15 ng of IL-10 per milligram of MCMs over the entire 17 day period (Fig. 2c).

### Cytokine levels

#### IL-10

**Fig. 3:**
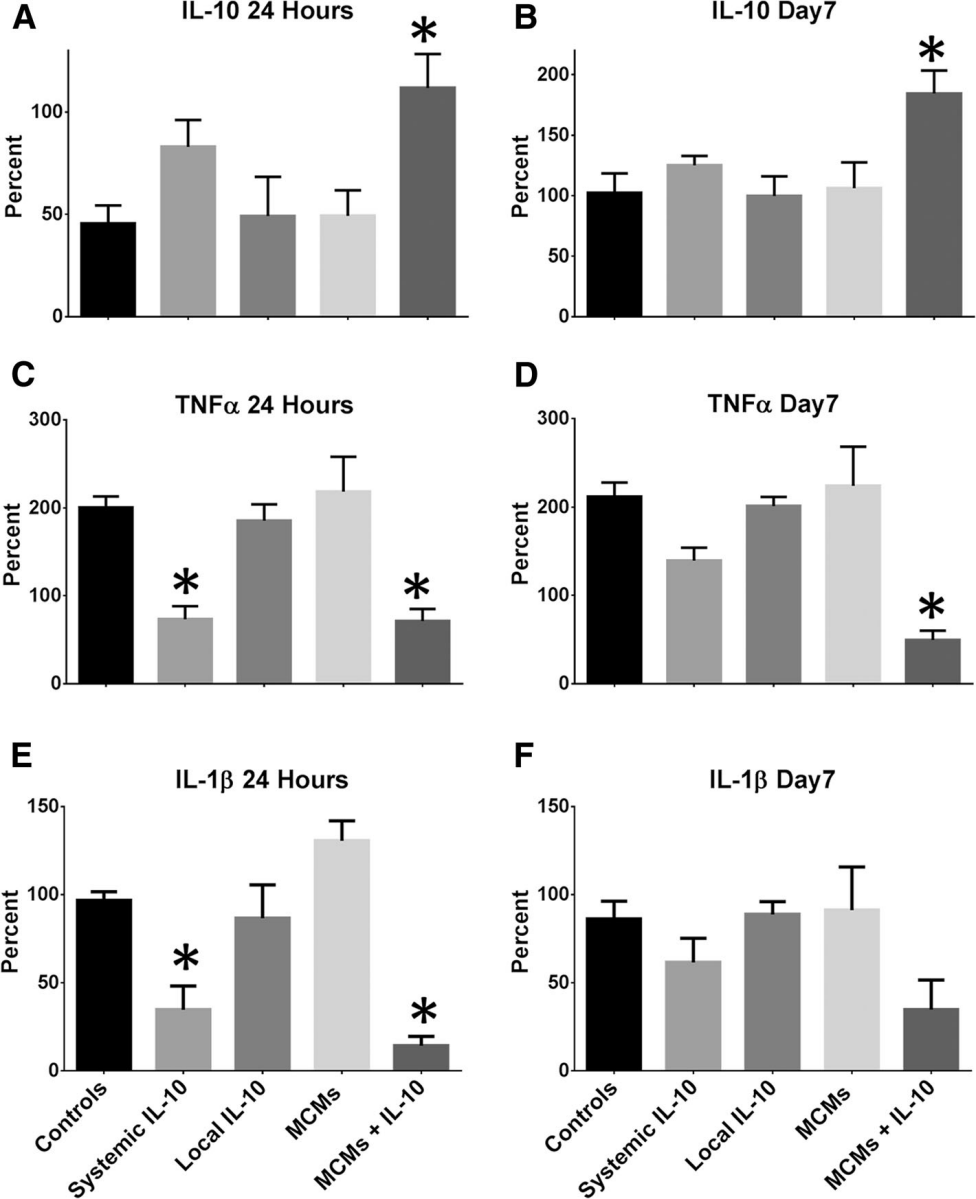
Assessment of cytokine levels. Spinal cords were harvested at 24 h and 7 days post-SCI and ELISAs were used to quantify IL-10 (**a**, **b**), TNFα (**c**, **d**), and IL-1β (**e**, **f**) in the injury site at each time point. The MCM+IL-10 treated rats had significantly higher levels of IL-10 at both 24 h and 7 days than the Controls, Local IL-10, and MCMs. Rats with the MCM+IL-10 and Systemic IL-10 treatments had less TNFα and IL-1β than the Controls, Local IL-10, and MCMs at 24 h. There was no significant difference in IL-1β levels at 7 days. All data is presented as percent increase from uninjured rats. *P < 0.05 in reference to Controls, Local IL-10, and MCMs (Tukey’s Test); error bars represent ± SEM; *n* = 7 for each time point

At 24 h post-SCI, there was a significant difference in IL-10 expression between the groups (F_4, 30_ = 3.972, *P* = 0.015, ANOVA; Fig. 3a). MCMs+IL-10 had a 111.6 ± 16.63% increase in IL-10 as compared to uninjured rats. This was significantly higher than the Controls at 45.5 ± 8.83% (*P =* 0.0235), Local IL-10 at 49.14 ± 19.14% (*P =* 0.0359), and MCMs at 49.20 ± 12.57% (*P =* 0.0361), all as the percent increase from uninjured rats. Systemic IL-10 was 82.99 ± 13.18%, which was not significantly different than the MCMs+IL-10 treatment (*P =* 0.6349).

Seven days post-SCI, there was also a significant difference between groups (F_4, 30_ = 4.527, *P =* 0.0056, ANOVA; Fig. 3b). MCMs+IL-10 had an even higher percent increase of IL-10, with 184.7 ± 18.70% increase from uninjured rats. MCMs+IL-10 still had significantly higher IL-10 than the Controls at 102.4 ± 16.08% (*P =* 0.0124), Local IL-10 at 99.74 ± 16.35% (*P =* 0.0094), and MCMs at 106.2 ± 21.44% (*P =* 0.0185), as percent increases from uninjured rats. Systemic IL-10 at a 125.1 ± 7.81% increase from uninjured rats was not significantly different than MCMs+IL-10 (*P =* 0.1128).

#### TNFα

At 24 h post-SCI, there was a significant difference in TNFα levels between groups (F_4, 30_ = 10.21, *P =* 0.0001, ANOVA; Fig. 3c). MCMs+IL-10 and Systemic IL-10 had the lowest TNFα percent increases, at 71.43 ± 13.88% and 73.57 ± 14.90%, respectively, as percent increases from uninjured rats. There was no significant difference between MCMs+IL-10 and Systemic IL-10 (*P =* 0.9999). MCMs+IL-10 and Systemic IL-10 had significantly less TNFα than the Controls at 200.4 ± 12.77% (*P*_MCMs + IL-10_ = 0.0027, *P*_SystemicIL-10_ = 0.0033), Local IL-10 at 185.5 ± 18.60% (*P*_MCMs + IL-10_ = 0.0093, *P*_SystemicIL-10_ = 0.0110), and MCMs at 218.6 ± 39.86% (*P*_MCMs + IL-10_ = 0.0006, *P*_SystemicIL-10_ = 0.0007), all shown as percent increases from uninjured rats.

At seven days post-SCI, there was also a significant difference between groups in TNFα levels (F_4, 30_ = 9.810, *P <* 0.0001, ANOVA; Fig. 3d). MCMs+IL-10 still had significantly lower TNFα concentrations than the Controls at 211.7 ± 16.09% (*P =* 0.0002), Local IL-10 at 201.1 ± 10.47% (*P =* 0.0006), and MCMs at 224.1 ± 44.43% (*P =* 0.0001) but Systemic IL-10 did not, as it increased to 139.3 ± 14.66% (*P*_Controls_ = 0.2013, *P*_LocalIL-10_ = 0.3435, and *P*_MCMs_ = 0.0967), with all results depicted as the percent increase from uninjured rats. Despite this difference, Systemic IL-10 and MCMs+IL-10 did not have significantly different TNFα concentrations (*P =* 0.0707).

#### IL-1β

At 24 h post-SCI, there was a significant difference between groups (F_4, 30_ = 15.44, *P =* 0.0001, ANOVA; Fig. 3e). Similar to TNFα at 24 h post-SCI, MCMs+IL-10 and Systemic IL-10 had significantly less IL-1β than the Controls at 96.6 ± 4.99% (*P*_MCMs + IL-10_ = 0.0003, *P*_SystemicIL-10_ = 0.0084), Local IL-10 at 86.52 ± 19.01% (*P*_MCMs + IL-10_ = 0.0017, *P*_SystemicIL-10_ = 0.0362), and MCMs at 130.6 ± 11.34% (*P*_MCMs + IL-10_ < 0.0001, *P*_SystemicIL-10_ < 0.0001), with all results depicted as the percent increase from uninjured rats. MCMs+IL-10 and Systemic IL-10 did not vary significantly with expression at 14.32 ± 5.273% and 34.67 ± 13.54%, respectively, as percent increases from uninjured rats (*P =* 0.7547). At 7 days post-SCI, there were no significant differences of IL-1β between groups (F_4, 30_ = 2.366, *P =* 0.0752, ANOVA; Fig. 3f).

### Macrophage phenotype

#### Flow cytometry

**Fig. 4:**
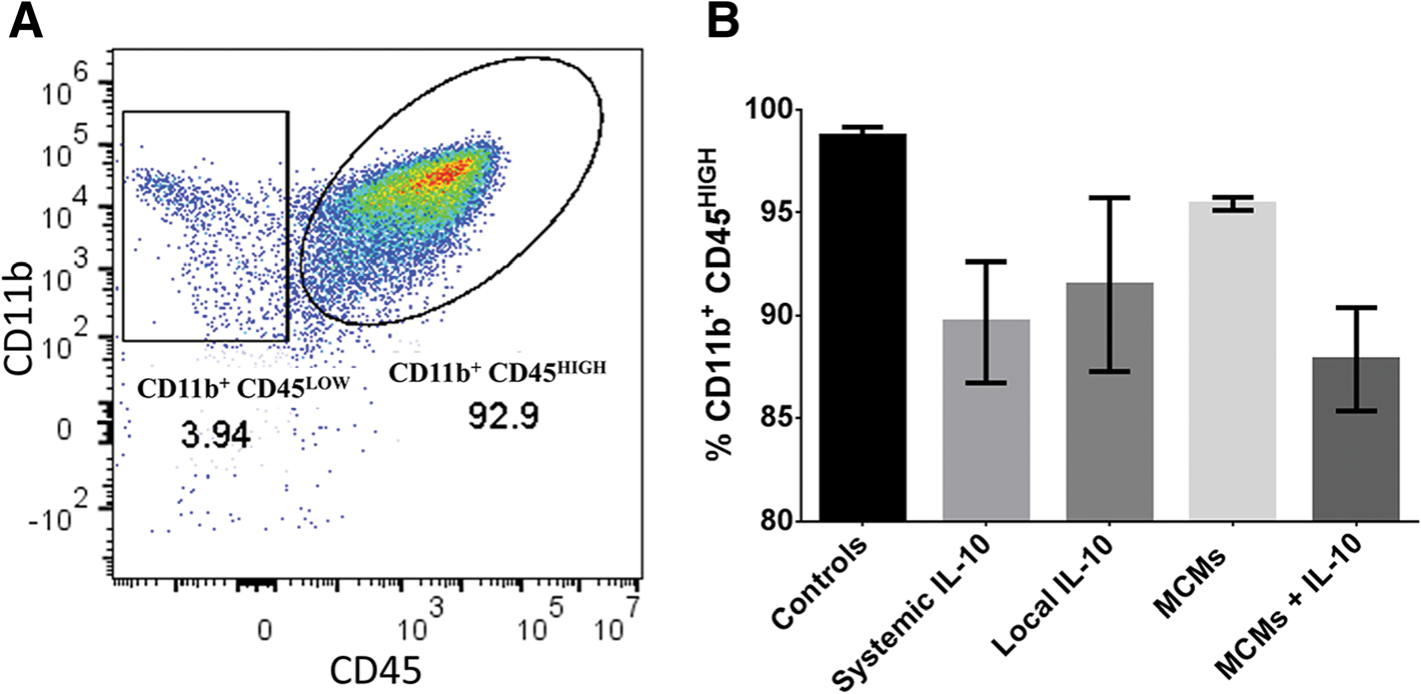
Flow cytometry performed seven days post-injury. Activated macrophages were distinguished by their expression of CD11b and CD45 (**a**). No difference was observed in the number of CD11b^+^CD45^HIGH^ macrophages between treatment groups (**b**). *P < 0.05 (Tukey’s Test); error bars represent ± SEM; *n* = 3 for Controls and 4 for all other groups

Seven days after injury, spinal cords were minced, cells were disassociated then stained with antibodies to recognize all macrophages (CD45 and CD11b), M1-specific macrophages (CD68 and CD80), M1 or M2b macrophages (CD86), and M2c macrophages (CD163). There was no significant difference in the number of CD11b^+^ CD45^HIGH^ cells between groups (F_4, 14_ = 2.392, *P* = 0.1003, ANOVA; Fig. 4).

**Fig. 5:**
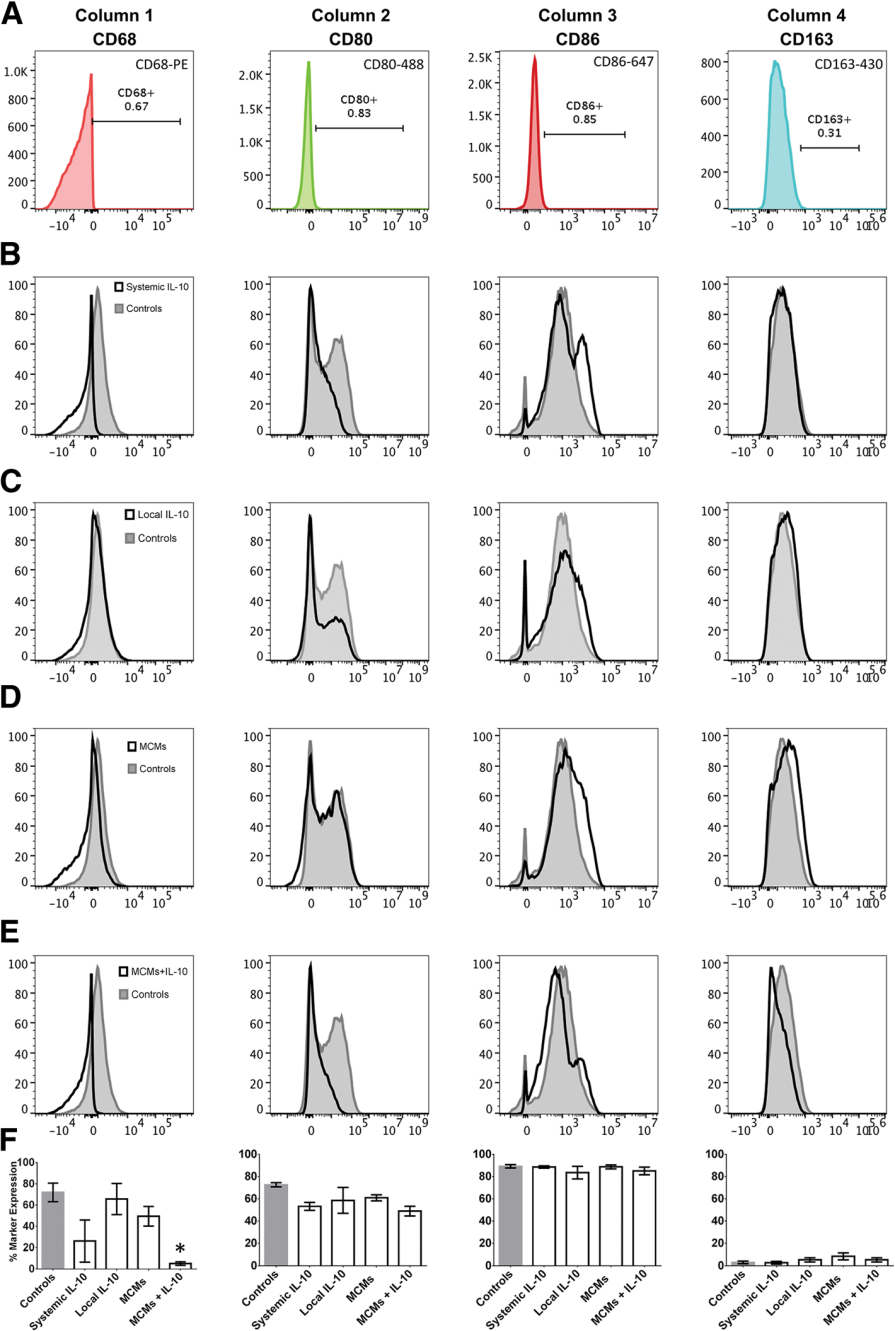
Macrophages analyzed for M1 and M2 antigens. The gates for M1 specific markers CD68, CD80, M1 or M2b marker CD86, and M2c marker CD163 were set using fluorescence minus one (FMO) (**a**). Each column represents one antigen. Representative histograms of CD68, CD80, CD86, and CD163 for Controls compared to Systemic IL-10 (**b**), Local IL-10 (**c**), MCMs (**d**), MCMs+IL-10 (**e**) are displayed. The percentages of antigen expression were quantified (**f**). *P < 0.05 (Dunnett’s Test); error bars represent ± SEM; *n* = 3 for Controls and 4 for all other groups

The expression of CD68 was significantly different between groups (F_4, 14_ = 4.624, *P* = 0.0137, ANOVA; Fig. 5 Column 1). MCMs+IL-10 had significantly less CD68 expression as compared to Controls (*P* = 0.0121), while no other group had a significant difference as compared to Controls (*P*_Systemic IL-10_ = 0.0960, *P*_Local IL-10_ = 0.9918, *P*_MCMs_ = 0.6003). There was no difference observed in expression of CD80 (F_4, 14_ = 1.822, *P* = 0.1806, ANOVA; Fig. 5 Column 2), CD86 (F_4, 14_ = 0.5947, *P* = 0.6723, ANOVA; Fig. 5 Column 3), or CD163 (F_4, 14_ = 1.215, *P* = 0.3481, ANOVA; Fig. 5 Column 4). All CD11b^+^ CD45^HIGH^ cells were futher analyzed for macrophage phenotype.

**Fig. 6:**
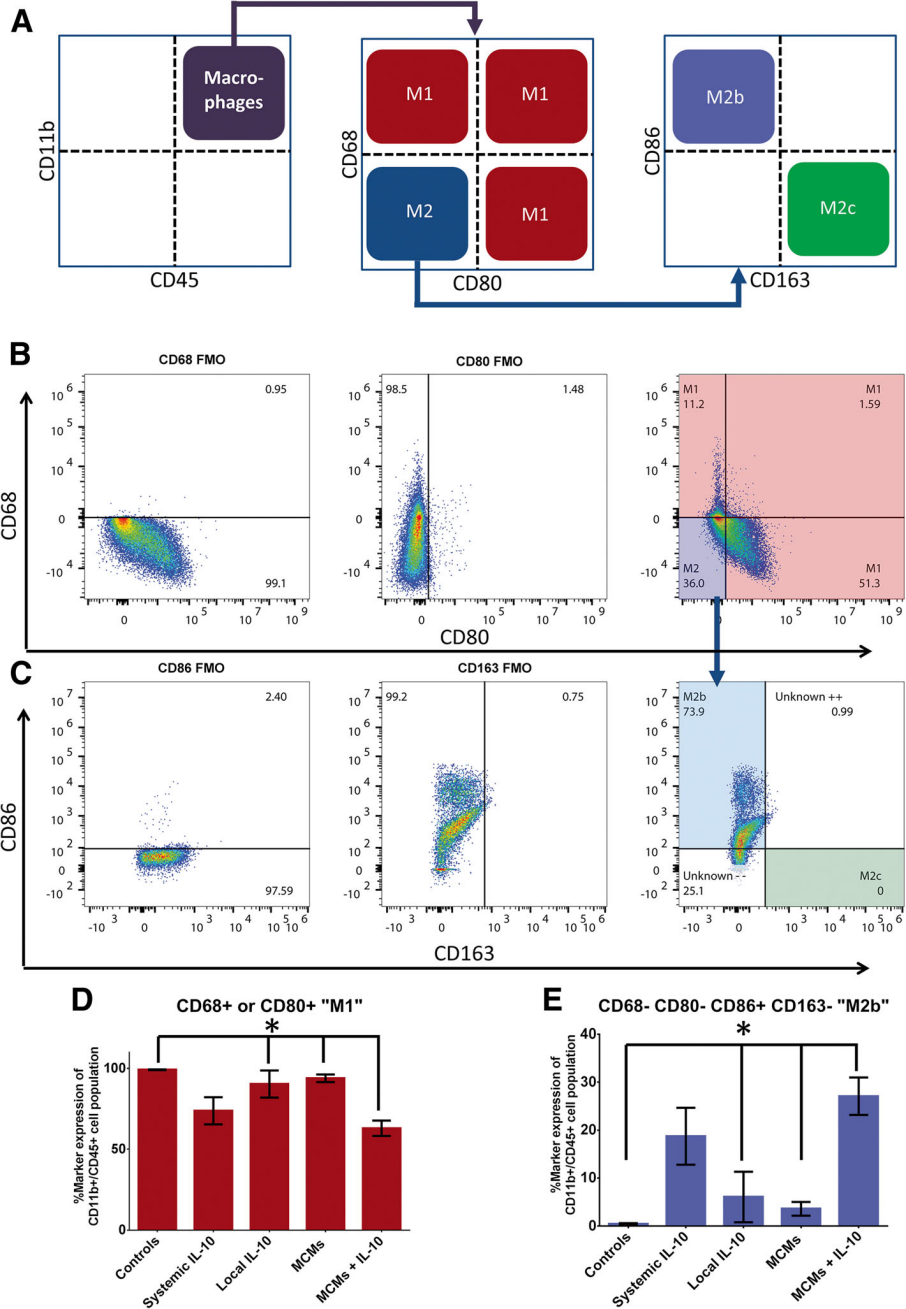
Percentages of M1 and M2 macrophages. The cells determined to be CD11b^+^CD45^HIGH^ macrophages were further analyzed for macrophage phenotype. Cells that were positive for the M1 markers CD68 or CD80 were characterized as “M1” macrophages and the CD68^−^CD80^−^ macrophages were further analyzed with CD86 and CD163 to distinguish early stage“M2b” and later stage “M2c,” as shown in the schematic (**a**). The FMOs for CD68 and CD80 were used to set the gates and a representative sample of MCMs+IL-10 is shown for the CD68 vs CD80 (**b**). The FMOs for CD86 and CD163 were used to set the gates for distinguishing “M2b” and “M2c” cells and the CD68^−^CD80^−^ “M2” macrophages were further analyzed for CD86 vs CD163 (**c**). Comparing across groups, MCMs+IL-10 had significantly less “M1” macrophages than the Controls, Local IL-10, and MCMs (**d**). MCMs+IL-10 also had significantly more “M2b” macrophages than the Controls, Local IL-10, and MCMs (**e**). *P < 0.05 (Tukey’s Test); error bars represent ± SEM; *n* = 3 for Controls and 4 for all other groups

For distinction throughout this paper, macrophages (CD11b^+^CD45^HIGH^) that were CD68^+^ or CD80^+^ were considered “M1,” those that were CD68^−^CD80^−^CD86^+^CD163^−^ were considered “M2b,” and those that were CD68^−^CD80^−^CD86^−^CD163^+^ were considered “M2c.” CD86 is expressed on both M1 and M2b macrophages; however, here, macrophages without co-expression of other M1 markers were considered to be M2b [1, 30–32]. There was a significant difference between groups in the percentage of cells that were “M1” (F_4, 14_ = 5.889, *P* = 0.0054, ANOVA; Fig. 6d) and “M2b” (F_4, 14_ = 6.958, *P* = 0.0027, ANOVA; Fig. 6e); however, the percentage of cells that were considered as a later stage “M2c” was negligible for all groups. MCMs+IL-10 had significantly less “M1” cells than Controls (*P* = 0.0109), Local IL-10 (*P* = 0.0421), and MCMs (*P* = 0.0193). Systemic IL-10 was not found to be significantly different than Controls (*P* = 0.0942) or any other treatment group. MCMs+IL-10 had significantly more “M2b” cells than Controls (*P* = 0.0067), Local IL-10 (*P* = 0.0210), and MCMs (*P* = 0.0095), while Systemic IL-10 did not reach significance against Controls (*P* = 0.0763) or any other treatment group.

#### Immunohistochemistry

**Fig. 7:**
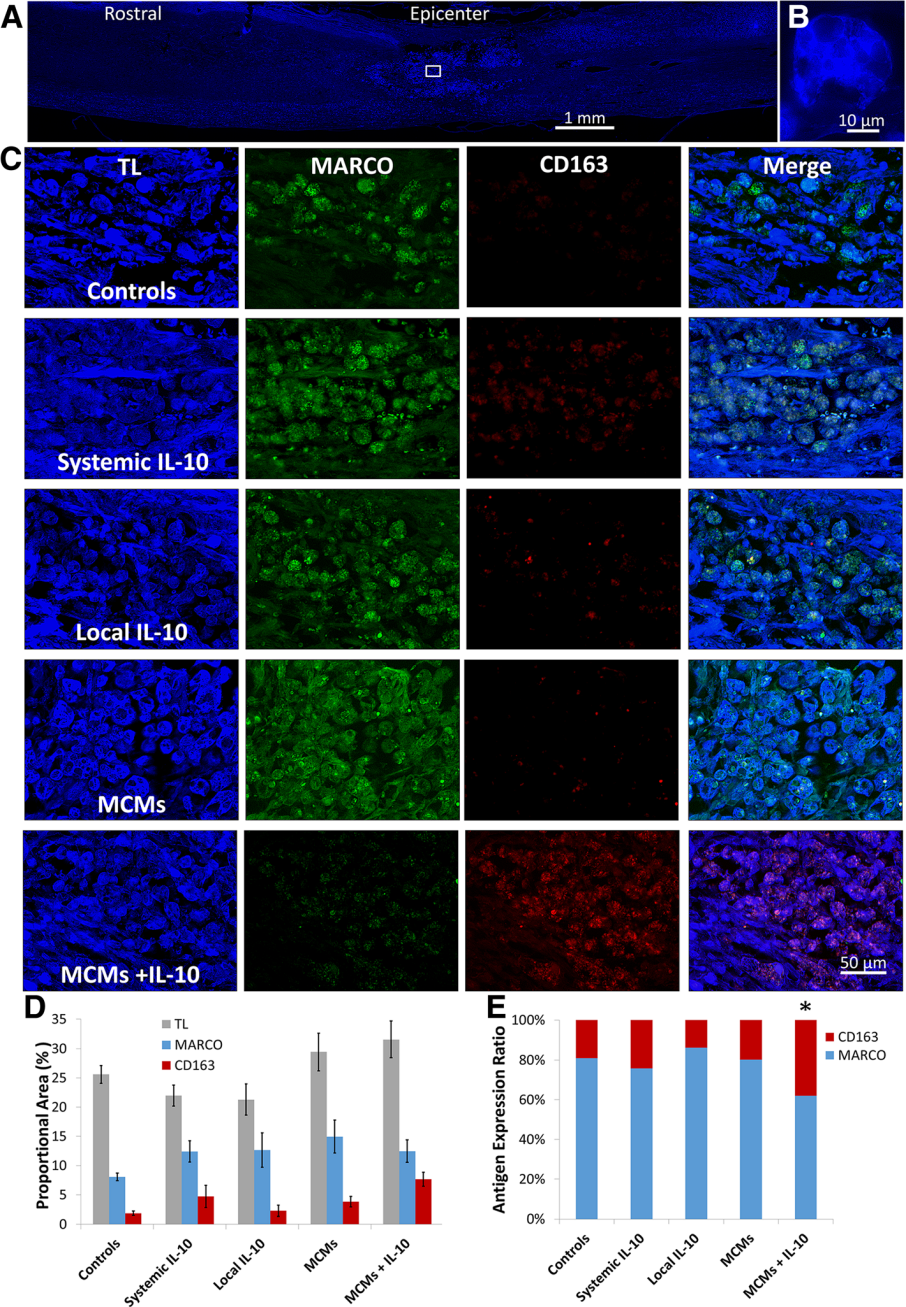
Histological analysis of IL-10 effect on macrophage phenotype. Sagittal sections of spinal cord containing the injury site were sectioned 10 μm thick and stained for all macrophages (TL), M1 macrophages (MARCO), and M2c macrophages (CD163) 7 weeks post-injury. TL stained sagittal section from a Control rat displaying the numerous macrophages labeled in the injury site (**a**) and a high magnification image showing the amoeboid form of an individual macrophage (**b**). Box shown in (**a)** is the location in the epicenter of the injury where representative micrographs of each group were taken (**c**). The area of tissue stained for macrophages and each phenotype were measured (**d**) and the ratio of MARCO to CD163 present in each group was calculated (**e**). The ratio of CD163 to MARCO was significantly higher in the MCMs+IL-10 group when compared to Controls, Local IL-10, and MCMs. *P < 0.05 (Tukey’s Test); error bars represent ± SEM; *n* = 5

The sagittal spinal cord segments were sectioned 10-μm thick and stained for M1 and M2 macrophage antigens, MARCO and CD163, respectively (Fig. 7a-b). There was a significant difference in the ratio of macrophages between the groups (F_4, 20_ = 6.374, *P =* 0.0018, ANOVA; Fig. 7d). MCMs+IL-10 were found to have a significantly higher expression of CD163 at 37.96 ± 0.89% as compared to the Controls at 19.05 ± 2.15% (*P =* 0.0113), Local IL-10 at 13.84 ± 3.81% (*P* = 0.0011), and MCMs at 19.84 ± 2.03% (*P* = 0.0158). Systemic IL-10 had 24.26 ± 6.440% CD163 expression but did not significantly vary from the Controls (*P* = 0.8450), Local IL-10 (*P* = 0.2870), MCMs (*P* = 0.9071), or MCMs+IL-10 (*P* = 0.0942) (Fig. 7c-d).

### Functional testing

**Fig. 8:**
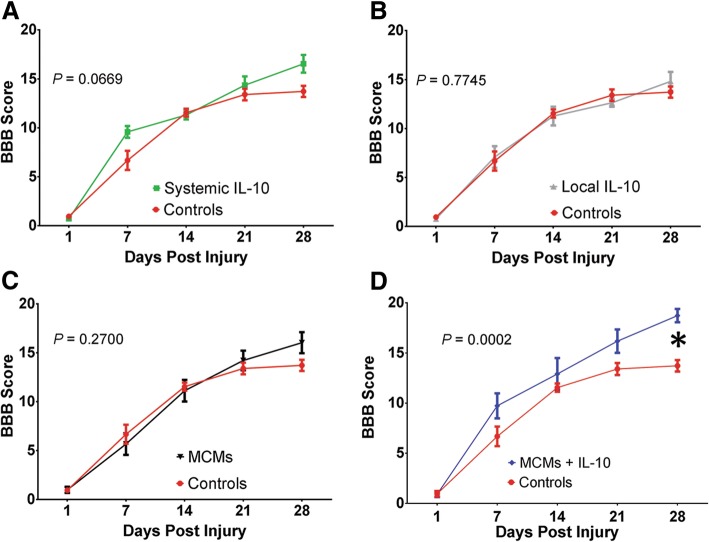
Assessment of functional recovery. Twenty-eight days post-SCI, BBB scores from the Systemic IL-10 group approached significance when compared to Controls (**a**), while rats in the Local IL-10 and MCMs groups were not significantly different from Controls (**b, c**). MCMs+IL-10 was the only group with significantly higher BBB scores than Controls (**d**). *P < 0.05 (Dunnett’s Test); error bars represent ± SEM; *n* = 10

On post-operation day one, all the rats’ BBB scores had dropped to a score of 0 or 1. During the 28-day testing period, two Control rats, one Systemic IL-10 rat, two local IL-10 rats, one MCM rat, and one MCM+IL-10 rat were treated for a urinary tract infection. There were no recurrent urinary tract infections. At the end of the 28-day period, improvement in functional scores was seen across all groups. The average BBB scores on day 28 were 13.73 ± 0.57 for the Controls, 16.55 ± 0.91 for Systemic IL-10, 14.82 ± 0.97 for Local IL-10, 16.05 ± 1.08 for MCMs, and 18.73 ± 0.66 for MCMs+IL-10. A two-way ANOVA determined there was a significant difference between the groups’ BBB scores (F_4,250_ = 7.315, *P* < 0.0001, ANOVA). When compared to the Controls on day 28, only the MCMs+IL-10 group had significantly higher BBB scores (*P*_Systemic IL-10_ = 0.0669, *P*_Local IL-10_ = 0.7745, *P*_MCMs_ = 0.2700, *P*_MCMs + IL-10_ = 0.0002 Fig. 8a–c).

### Axon sparing

**Fig. 9:**
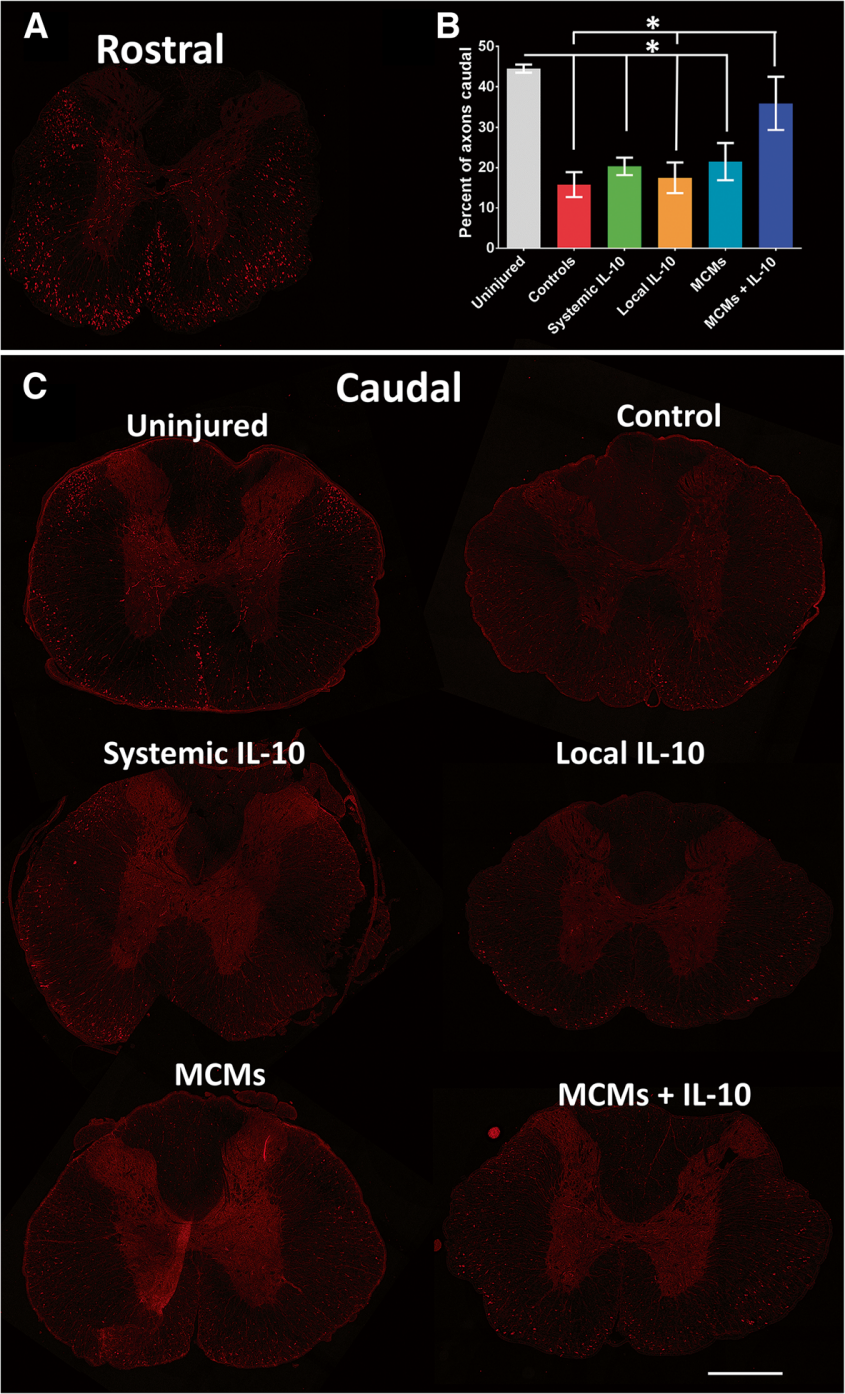
Axon tracing in brainstem tracts. Twenty-eight days post-SCI, rats were injected with BDA in the red nucleus and reticular formation and harvested 21 days after injection. Transverse sections displayed are rostral to the injury site (**a**), caudal in an uninjured rat, Control, Systemic IL-10, Local IL-10, MCMs, and MCMs+IL-10 (**c**). The percentage of axons that passed through to the caudal sections was calculated (**b**). The BDA injected rats had an average of 977 ± 93 axons labeled rostral to the injury. Uninjured rats had a significantly higher number of axons extending through to the caudal sections compared to all groups except the MCMs+IL-10 group. The rats treated with MCMs+IL-10 had a significantly higher percentage of labeled axons caudal to the injury, 35.92 ± 6.559%, when compared to Controls 15.81 ± 3.081% and Local IL-10 17.50 ± 3.804%. Red represents BDA labeled axons; blue represents DAPI; *P < 0.05 (Tukey’s Test); error bars represent ± SEM; *n* = 4 per group; scale bar = 500 μm

The rubrospinal and reticulospinal tracts were analyzed together as a single entity. The BDA was taken up well throughout all groups, with 955 ± 123 axons labeled rostral to the injury. There was a significant difference in the percent of labeled axons that passed through the injury site between the groups (F_5,18_ = 8.539, *P =* 0.0003, ANOVA). The uninjured rats showed 44.49% ± 1.02% of the axons rostral are still present caudal to T10. The Controls presented with an average of 15.81 ± 3.08%, the Systemic IL-10 group with 20.33 ± 2.17%, the Local IL-10 group with 17.50 ± 3.80%, the MCMs group with 21.50 ± 4.61%, and the MCMs+IL-10 group with 35.92 ± 6.55% of axons crossing the injury site (Fig. 9). A significant difference was found when the percent of axons caudal to the injury of the MCMs+IL-10 group was compared to that of the Controls (*P =* 0.0216) and Local IL-10 (*P* = 0.0399). No other comparisons of groups with contused rats reached significance. Though the percent of axons caudal to the injury of the uninjured rats was significantly greater than the Controls (*P* = 0.0009), Systemic IL-10 (*P* = 0.0047), Local IL-10 (*P* = 0.0016), and MCMs (*P* = 0.0074), the uninjured rats did not reach significance against MCMs+IL-10 (*P* = 0.6503).

**Fig. 10:**
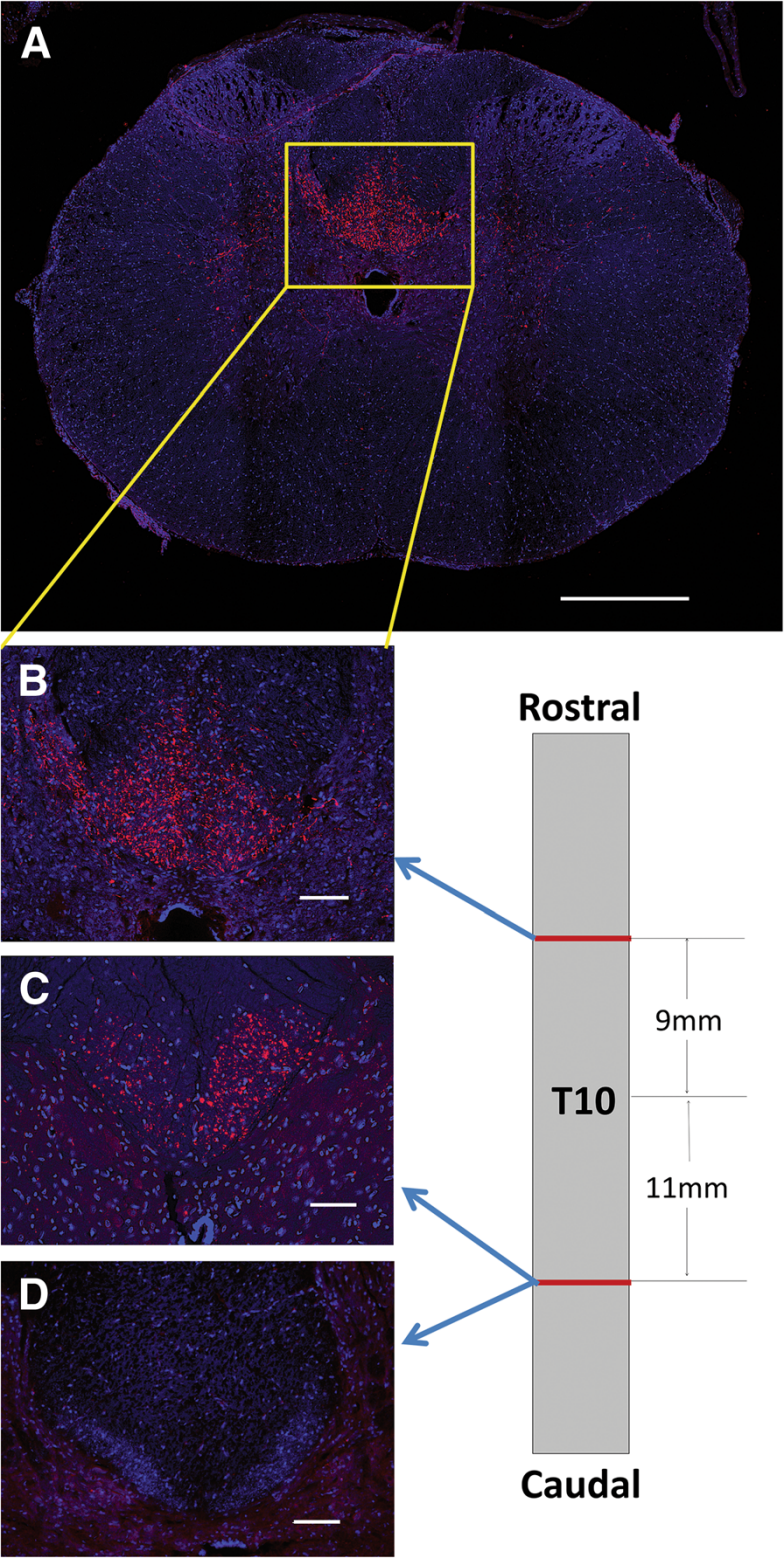
Axon tracing in the corticospinal tracts. Twenty-eight days post-SCI, rats were injected with BDA in the motor cortex and harvested 21 days after injection. Transverse sections rostral to the T10 level had an average of 739 ± 58 labeled axons in the dorsal corticospinal tract (**a**, **b**) and uninjured rats had an average of 97 ± 56 in the region caudal to the T10 level (**c**), which was significantly more than all other groups (*P* < 0.0001). Virtually no axons were labeled in the dorsal corticospinal tract caudal to the injury site in Controls (**d**) or any of the treatment groups and there was no significant difference between any of the groups that underwent an SCI. Red represents BDA labeled axons; blue represents DAPI; *n* = 3 per group; scale bars = 500 μm (**a**), 100 μm (**b**–**d**)

The BDA was also taken up well by the motor cortex neurons and there were hundreds of labeled axons rostral to the injury. Although 726 ± 64 labeled axons were present in the dorsal corticospinal tract rostral to the injury, virtually no axons crossed the injury site (Fig. 10b, d). Thus, the uninjured rats presented with significantly more dorsal corticospinal tract axons caudal to T10 as compared to all contused groups (F_5,12_ = 55.22, *P <* 0.0001, ANOVA), with 34.03 ± 4.57% and less than 1%, respectively (*P* < 0.0001 for all contused groups vs. uninjured rats). The lateral corticospinal tract expressed on each side an average of 32 ± 4 axons rostral. The uninjured rats had significantly more lateral corticospinal tract axons caudal to T10 than all contused groups (F_5,12_ = 32.08, *P* < 0.0001, ANOVA), with 59.64 ± 3.7% and fewer than 10%, respectively (*P* < 0.0001 for all contused groups vs. uninjured rats). The ventral corticospinal tract presented with an average of 17 ± 0.5 axons rostral to the injury. The uninjured rats presented with significantly more ventral corticospinal tract axons caudal to T10 than all contused groups (F_5,12_ = 6.154, P = 0.0047, ANOVA), with 33.19 ± 3.7% and fewer than 10%, respectively (*P* < 0.05 for all contused group vs. uninjured rat comparisons). There were no significant differences between any of the contused groups in any of the corticospinal tracts.

### Electrophysiology

**Fig. 11:**
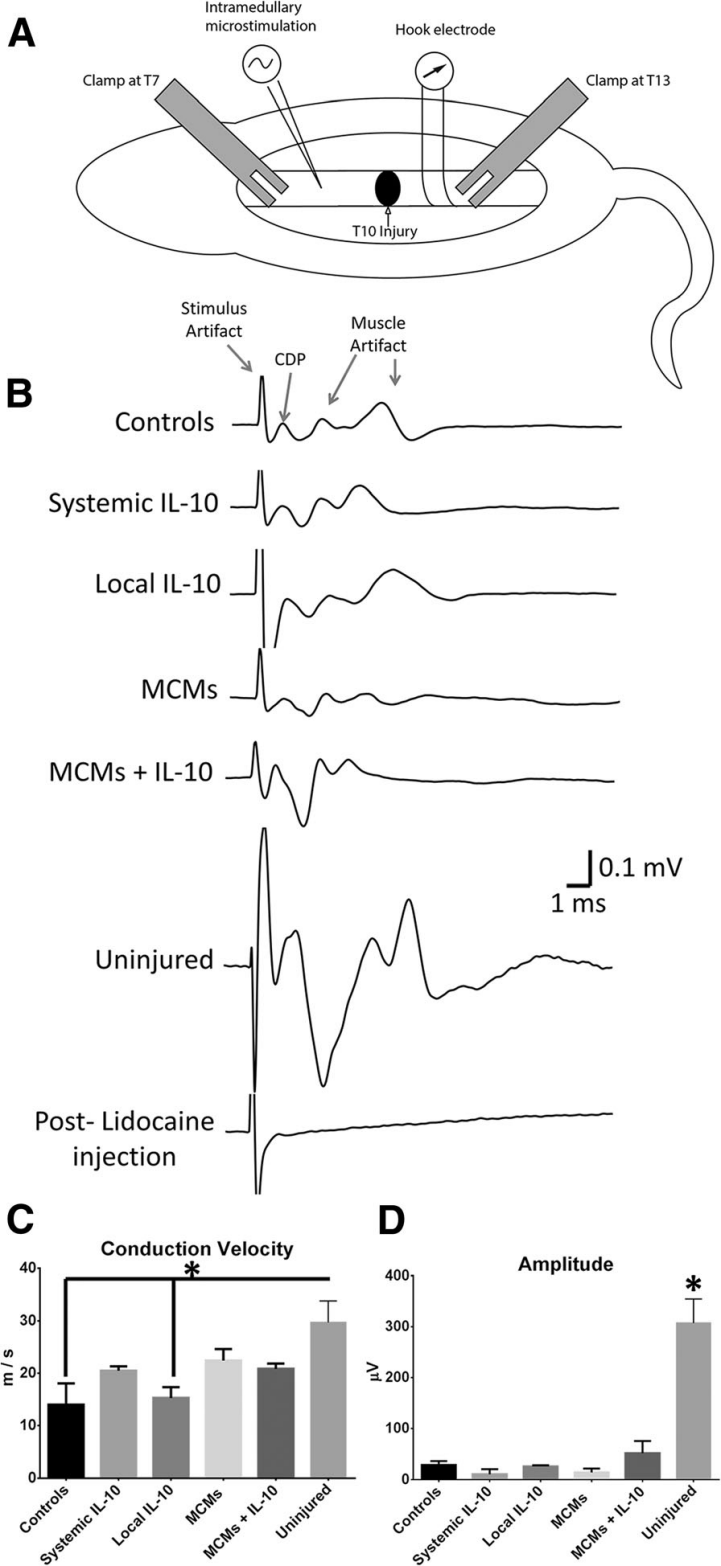
Analysis of nerve conduction across the injury site via electrophysiology. A stimulating electrode was placed in the spinal cord rostral to the injury site and a recording hook electrode was placed on the dorsal side of the spinal cord caudal to the injury (**a**). The cord dorsum potential was measured and a representative result is displayed from all groups, with the signal completely removed after an injection of lidocaine (**b**). The conduction velocity was significantly better in the uninjured rats compared to Controls and Local IL-10 groups (**c**), and the amplitude was significantly larger in uninjured rats compared to all other groups (**d**). There was no significant difference between any of the groups that underwent a SCI in amplitude or conduction velocity. Error bars represent ± SEM. *P < 0.05 (Tukey’s Test); error bars represent ± SEM; *n* = 3

At seven weeks post-SCI, nerve conduction was tested across the injury site. A stimulating electrode was placed rostral to the injury with the recording hook electrode placed caudal to the injury (Fig. 11a). The CDP was consistently recorded and after an injection of lidocaine the signal was completely removed (Fig. 11b). MCMs+IL-10 had a mean CDP amplitude of 51.45 ± 23.95 μV, which was larger than the Controls at 28.44 ± 7.56 μV; however, significance was not reached between any contused groups. The uninjured rats had a significantly higher amplitude of 307.3 ± 47.25 μV (F_5, 12_ = 26.77, *P* < 0.0001, ANOVA, Fig. 11c) as compared to all contused groups (*P* < 0.0001 for all contused group vs. uninjured rats comparison). There was also no significant difference in conduction velocity between contused groups. The uninjured rats had a significantly faster conduction velocity of 29.74 ± 4.11 m/s (F_5, 12_ = 4.284, *P =* 0.0181, ANOVA) than the Controls at 13.97 ± 4.11 m/s (*P* = 0.0146) and Local IL-10 at 15.26 ± 2.10 m/s (*P* = 0.0257) but did not reach significance against Systemic IL-10 at 20.50 ± 0.84 m/s, MCMs at 22.44 ± 2.19 m/s and MCMs+IL-10 at 20.85 ± 1.00 m/s (Fig. 11d).

### Lesion size

**Fig. 12:**
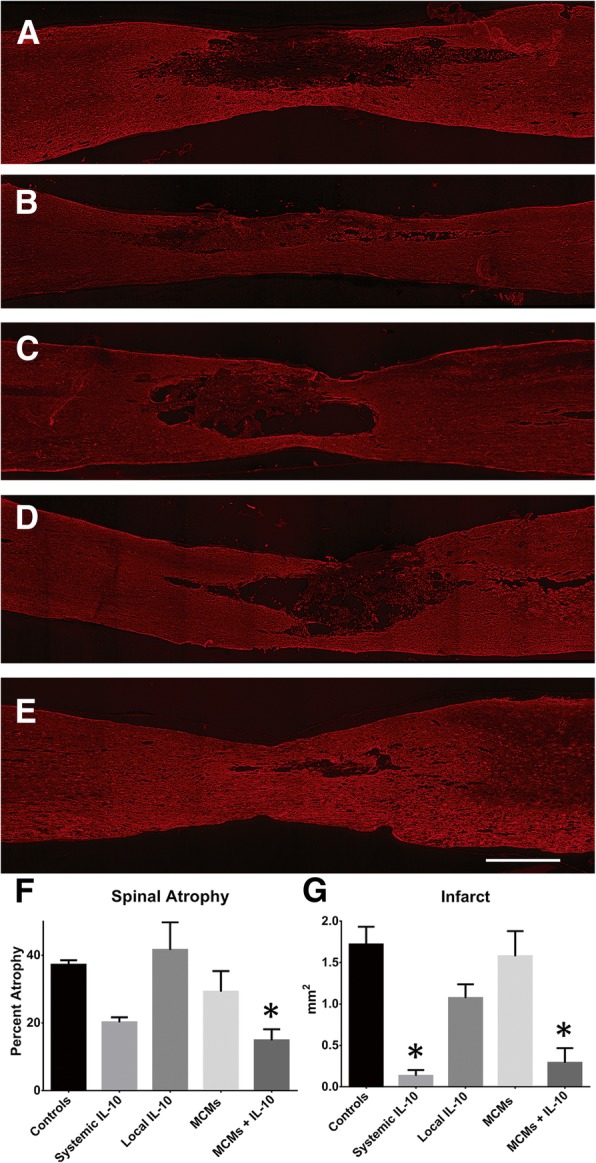
Analysis of IL-10 effect on lesion size and spinal atrophy. Sagittal sections of spinal cord containing the injury site were sectioned 10 μm thick and GFAP positive astrocytes were labeled in Controls (**a**), Systemic IL-10 (**b**), Local IL-10 (**c**), MCMs (**d**), and MCMs+IL-10 (**e**). The spinal cord thickness was measured 5 mm rostral to the epicenter, at the epicenter, and 5 mm caudal to the epicenter and the percent in reduced thickness at the injury site was calculated (**f**). The MCMs+IL-10 was the only group with significantly less spinal atrophy than Controls. The infarct was measured as the area of tissue outlined by reactive astrocytes (**g**). Both the Systemic IL-10 and MCMs+IL-10 had a significantly smaller infarct area when compared to the Controls. *P < 0.05 (Dunnett’s Test); error bars represent ± SEM; *n* = 4; scale bar = 1 mm

Sagittal sections containing the lesion are presented with representatives from each group (Fig. 12a–e). There was a significant difference between the groups with regard to both percent of spinal atrophy (F_4,15_ = 5.272, *P =* 0.0074, ANOVA; Fig. 12f) and area of infarct (F_4,15_ = 13.04, *P* < 0.0001, ANOVA, Fig. 12g). There was significantly less spinal atrophy in the MCMs+IL-10 group than the Controls (*P* = 0.0185). There was no significant difference in atrophy for the Systemic IL-10, Local IL-10, or the MCMs (*P*_Systemic IL-10_ = 0.0813, *P*_Local IL-10_ = 0.9196, *P*_MCMs_ = 0.6125; Fig. 12f). Both MCMs+IL-10 and Systemic IL-10 had significantly smaller area of infarct than the Control group (*P*_MCMs+IL-10_ = 0.0005, *P*_Systemic IL-10_ = 0.0002; Fig. 12g). There was no significant difference in infarct size for Local IL-10 or the MCMs when compared against the Controls (*P*_Local IL-10_ = 0.1140, *P*_MCMs_ = 0.9615; Fig. 12g).

## Discussion

As shown previously, mineral coatings have a high protein-binding propensity and the capability to release proteins over a controllable time frame with high levels of protein biological activity [18–20, 33–36]. In this study, we hypothesized a local sustained release of IL-10 from mineral coatings would reduce the prolonged inflammation that occurs after SCI. The in vitro release profile indicates an initial burst of IL-10 for 2 days, followed by a continuous release of IL-10 for at least 17 days, evidence that the coating has the ability to maintain protein delivery for a length of time through the initial inflammatory period post-SCI [2, 3].

### IL-10’s effect on inflammation

Normal wound healing in skin or muscle occurs over three defined stages: inflammation, proliferation, and remodeling. Throughout healing, macrophages exist on a continuum from M1 inflammatory macrophages to M2 anti-inflammatory macrophages dictated by the extracellular environment. In the inflammatory stage, M1 macrophages aid the innate immunity by inducing inflammation through the production of TNFα and IL-1β, while phagocytosing bacteria and debris [1, 37]. As proliferation begins, M1 macrophages gradually change phenotype to a variety of M2 macrophage subsets with different functions. Generally, M2 macrophages attenuate the production of pro-inflammatory cytokines and reactive oxygen species (ROS), in addition to having tissue-repairing properties. The production of IL-10 is traditionally attributed to the M2b phenotype [31, 38] and acts in tissue remodeling during the proliferative stage of muscle and skin wound healing [8, 39]. During healing, M2c macrophages remain to aid in the remodeling phase [39]. However, defined stages of healing are not present post-SCI [1, 4]. Without proper termination of the inflammatory stage, the presence of activated macrophages (M1 and M2) may last for years [1, 39].

Post-SCI, the poor transition of macrophages complicates healing [39–41]. Binding of IL-10 to the IL-10 receptor activates the Jak/STAT pathway, which results in a reduction of pro-inflammatory cytokines released from macrophages/microglia [12]. One mechanism by which IL-10 treatment is thought to improve functional recovery post-SCI is through macrophage deactivation: hindering the production of pro-inflammatory cytokines prevents further damage to the spinal cord by attenuating cell death [42, 43] and blood–spinal cord barrier disruption [44], in addition to reducing ROS and neurotoxic substance production. These benefits have been observed in previous studies where IL-10 induced nerve growth factor production in astrocytes and reduced astrogliosis post-TBI in rats [6, 45].

In this study, the MCMs+IL-10 group observed a significant increase in IL-10 at both 24 h and 7 days post-SCI (Fig. 3a-b). In accordance with previous studies, the Systemic IL-10 treatment attenuated TNFα and IL-1β production at 24 h post-SCI, but failed to reach significance at 7 days post-SCI [14, 46, 47]. The MCMs+IL-10 group did significantly attenuate the production of TNFα at 7 days post-SCI, proving the sustained release of IL-10 effective. However, there was no significant difference in IL-1β production at 7 days post-SCI (Fig. 3f). In agreement with the cytokine analysis, MCMs+IL-10 observed a significant decrease in the number of “M1” macrophages as compared to Controls at 7 days post-SCI, evaluated with flow cytometry (Fig. 6d). Although there was no difference in the expression of the M2c marker CD163 between any of the groups 7 days post-SCI, there was a significant increase in the “M2b” population (CD11b^+^ CD45^HIGH^ CD68^−^ CD80^−^ CD86^+^ CD163^−^) in the MCM + IL-10 group (Fig. 6e). The sustained release of IL-10 is likely contributing to the decreased expression of M1 macrophage markers, but complete conversion to an M2c expressing CD163 was not observed at the 7-day time point. Immunohistochemistry analysis at 7 weeks post-SCI, however, did show a significant increase in the ratio of CD163:MARCO (M2c:M1) in MCMs+IL-10 as compared to Controls (Fig. 7c). Co-expression of MARCO and CD163 was observed, indicating that these macrophages lie between M1 and late stage M2 on the continuum but lean closer to M2 with the MCMs+IL-10 treatment as compared to Controls (Fig. 7). This is likely beneficial as M2c macrophages (CD163^+^) are found in the later stages of wound healing [1, 37, 39].

### Using IL-10 to treat SCI

IL-10 treatment has been shown to improve functional recovery in Sprague-Dawley rats post-SCI [13–15]. Here, the MCMs+IL-10 group had the highest functional score on day 28 and was the only group to reach significance when compared to the Controls (Fig. 8d). In addition, IL-10 treatment has previously reduced lesion size post-SCI [14, 48]. We observed similar reductions in lesion size, with a significant reduction in infarct size with both the Systemic IL-10 treatment and the MCMs+IL-10 treatment as compared to Controls. However, there was no significant difference between MCMs+IL-10 and Systemic IL-10 in atrophy (*P* = 0.9358; Fig. 12f) or infarct size (*P* = 0.9791; Fig. 12g).

The MCMs+IL-10 group also expressed the greatest percent of spared axons in the reticulospinal and rubrospinal tracts, which was the only group significantly higher than the Controls. Though functional recovery was observed across all groups, the dorsal corticospinal tract presented with virtually no axons crossing the injury site in any of the five contused groups, unlike the reticulospinal and rubrospinal tracts. This data, in conjunction with the functional recovery assessed through BBB scores, suggests changes involving spared axons may have occurred within the spinal cord to permit functional recovery.

The number of axons spared in the rubrospinal and reticulospinal tracts suggests a plastic capability of the rat central nervous system (CNS) which allows for heavier reliance on these tracts for motor function. Both the rubrospinal and reticulospinal tracts are known to participate in motor function in healthy models, specifically through limb movement, though to different capacities. The rubrospinal tract maintains greater control of more precise movements and the reticulospinal tract expresses control of larger, gross locomotor movement and rhythm [49–54]. It is possible that the CNS is utilizing the spared axons in these tracts, which already express an innate participation in locomotor function, to regain motor capabilities after injury. Animal models have shown the rubrospinal tract is capable of absorbing the role of the corticospinal tract (CST) in the event of injury [55–59]. Although the rubrospinal tract is well developed in lower mammals (i.e., cats and rats), its importance in upper mammals appears to have diminished with the evolutionary development of the CST [60]. This may hinder translation to human models, which do not express a prominent rubrospinal tract [61].

Another possible explanation is that spontaneous axonal sprouting may have occurred and aided in improving functional recovery in the MCM+IL-10 group. Previous studies have shown the ability of various tracts within the spinal cord to express axonal sprouting [55, 62–65]. The axon tracer used in this study, BDA, does not cross synapses. Thus if there was axonal sprouting and synapsing, it would not have been detected.

No significant difference was found between contused groups for conduction velocity or amplitude (Fig. 11c-d). With few axons extending through the injury site post-SCI, the CST likely created a very small signal in all the rats, regardless of treatment. The location of the rubrospinal and reticulospinal tracts, along with the placing of the recording electrode, may have impacted the results. The rubrospinal and reticulospinal tracts are located deep and laterally in the spinal cord. With the receiving electrode placed gently on the dorsal side of the spinal cord, voltage from action potentials in deeper tracts would be reduced due to distance.

## Conclusion

Following a SCI, diverse cellular and molecular responses result in abnormal environmental conditions, which both greatly hinder axon growth and result in further cell death. The present study demonstrates the use of MCMs loaded with IL-10 to provide a sustained release of the anti-inflammatory cytokine in order to establish a less severe environment and allow for axon sparing. The results are promising, with in vitro results expressing a release profile of IL-10 for at least 17 days. This extended release is carried over in vivo resulting in lower levels of TNFα and IL-1β, lower levels of “M1” macrophages, greater numbers of surviving axons, enhanced functional recovery, and a smaller lesion size in those rats treated with IL-10-bound MCMs. These outcomes exemplify the neuro-protective properties of IL-10 when delivered over a prolonged period of time. Interestingly, however, the lack of variation among the groups with regard to axon sparing in the corticospinal tracts calls for further studies, especially when taking into the account the considerable differences observed in the rubrospinal and reticulospinal tracts of the groups. Further investigations, which explore such topics as the release of multiple therapeutic proteins and the use of an axon tracer that has the ability to cross synapses, are necessary to better enhance our understanding.

## Abbreviations

AP: Anterior–posterior
BBB: Basso-Beattie-Bresnahan
BDA: Biotinylated dextran amine
BSA: Bovine serum albumin
CDP: Cord dorsum potential
CNS: Central nervous system
CST: Corticospinal tract
DAPI: 4′,6-diamidino-2-phenylindole
DV: Dorsal–ventral
EDS: Energy dispersive spectroscopy
ELISA: Enzyme-linked immuno-sorbent assay
FMO: Fluorescence minus one
GFAP: Glial fibrillary acidic protein
IL-1β: Interluekin-1 beta
IL-10: Interleukin-10
MARCO: Macrophage receptor with collagenous structure
MC: Motor cortex
MCMs: Mineral Coated Microparticles
ML: Medial–lateral
mSBF: Modified simulated body fluid
nDS: Normal donkey serum
PFA: Paraformaldehyde
RF: Reticular formation
RN: Red nucleus
ROS: Reactive oxygen species
SCI: Spinal Cord Injury
SEM: Standard Error of the Mean
TNFα: Tumor necrosis factor-alpha
β-TCP: β-tricalcium phosphate
